# Comparison of direct versus concentrated smear microscopy in detection of pulmonary tuberculosis

**DOI:** 10.1186/1756-0500-6-291

**Published:** 2013-07-25

**Authors:** Mohammad Khaja Mafij Uddin, Md Raihan Chowdhury, Shahriar Ahmed, Md Toufiq Rahman, Razia Khatun, Frank van Leth, Sayera Banu

**Affiliations:** 1 International Centre for Diarrhoeal Disease Research, Bangladesh; 2 Department of Global Health, Amsterdam Institute for Global Health and Development, Academic Medical Centre, University of Amsterdam, Amsterdam, The Netherlands

**Keywords:** Tuberculosis, Sensitivity, Direct vs. Concentrated smear microscopy, Sputum smear microscopy

## Abstract

**Background:**

Sputum smear microscopy is fast and inexpensive technique for detecting tuberculosis (TB) in high incidence areas but has low sensitivity. Physical and chemical sputum processing along with centrifugation have been found to show promise in overcoming this limitation. Our objective was to compare the sensitivity of smear microscopy obtained with smears made directly from respiratory specimens to those from concentrated specimens.

**Methods:**

By active screening, 915 TB suspects were identified from Dhaka Central Jail and sputum specimens were aseptically collected. Direct smears were prepared by taking a small portion of the purulent part of the sputum with a sterile loop. The specimens were then processed by a standard N-acetyl-L-cysteine-NaOH digestion-decontamination method to prepare concentrated specimens. Both smears were then air dried, heat fixed, and stained by the Ziehl-Neelsen staining technique. The stained slides were examined under oil immersion and were graded following International Union Against Tuberculosis and Lung Diseases guidelines. All the specimens were inoculated into Lowenstein-Jensen (L-J) media and culture results were considered as gold standard to calculate sensitivity.

**Results:**

Of 915 specimens, 73 (8%) specimens were positive both on direct and concentrated methods, one sample was positive on direct microscopy but was negative on concentrated method. An extra 14 (1.5%) samples were positive on concentrated method which were negative on direct smear. In L-J media 105 specimens were found positive for TB bacilli and of them, 74 (70.5%) and 87 (82.9%) were positive in direct and concentrated smear, respectively. The sensitivity of direct and concentrated smear microscopy was different when using positive culture as the gold standard (71% vs. 83%).

**Conclusions:**

The results showed that concentrated technique increases the sensitivity of microscopy up to 12%. Therefore, the national programs in high TB burden countries may consider incorporating the technique into their guidelines at least in the district and higher level laboratories to improve case finding strategy.

## Background

Tuberculosis (TB) continues to be a major public health threat worldwide despite the availability of many highly sensitive diagnostic tools and highly efficacious treatment for decades. It is more of a threat in the developing world. There were an estimated 8.8 million new cases (incidence) of TB (range, 8.5 million–9.2 million) globally in 2010, 1.1 million deaths (range, 0.9 million–1.2 million) among human immunodeficiency virus (HIV)-negative cases of TB and an additional 0.35 million deaths (range, 0.32 million–0.39 million) among people who were HIV-positive. This makes TB the second leading cause of death among the infectious diseases [1]. A staggering 95% of these cases and deaths occur in the developing countries [2,3].

Early diagnosis of TB is crucial both clinically and epidemiologically. It is essential to ensure proper and early identification of cases, and good treatment outcomes to be able to limit its transmission and obtain successful TB control [4]. The gold standard for pulmonary TB diagnosis is culture of sputum in liquid media. However, due to lack of access to culture facilities and the long turn-around times involved with sputum culture, most programmes use direct Ziehl-Neelsen (Z-N) microscopy for detection of acid-fast bacilli (AFB) in sputum smears as their main diagnostic tool. In this method, the sputum specimens are smeared directly on to the slides without any processing and subjected to ZN staining. AFB microscopy is believed to be the most practical and fastest technique in establishing a diagnosis of pulmonary TB, especially in developing countries where most of the TB cases live [4,5]. Studies have shown that direct smear microscopy is highly specific in settings where TB is more prevalent [6,7]. Though AFB microscopy is simple, inexpensive and provides rapid result, it has some limitations. The threshold for detection of AFB in sputum samples under optimal conditions is between 10^4^ and 10^5^ bacilli per ml. The sensitivity and specificity of AFB microscopy is low when compared to culture method. In some studies it has been shown that this technique has a low sensitivity, 22-43% for a single smear [8] and up to 60% under optimal conditions [9,10] when compared with that of cultures. Sensitivity is even more reduced if samples are of poor quality, which is often the case in children and HIV-coinfected patients [11,12]. Although all mycobacterial species are acid fast, this assay is highly specific for *M. tuberculosis* in countries where TB is endemic [13].

Microscopy clearly has many advantages when it comes to speed and feasibility, and if sensitivity could be improved it has the potential to become an even more valuable tool for National TB Control Programmes (NTPs) around the world. In the last decade many researchers have suggested that the performance of sputum smear microscopy can be significantly improved if sputum is liquefied with chemical reagents and then concentrated by centrifugation or sedimentation prior to acid-fast staining [14,15]. Out of them, the technique using N-acetyl-L-cysteine (NALC) with 2% sodium hydroxide (NaOH) is considered to be the best. NALC acts as a strong mucus digester and the smear processed by it has less debris and a greater concentration of AFB [16]. This method has been found to increase the sensitivity of microscopy substantially [10]. However, it requires some level of staff training, increases time needed for diagnosis, and requires some level of biosafety arrangement to ensure the security of the lab personnel. Due to resource constraints, it is not applied in the majority of TB laboratories in developing countries [17,18].

Another chemical, sodium hypochlorite, usually known as household bleach is considered as an ideal chemical processing agent for use in low income countries. It is widely available and inexpensive, and its disinfectant properties could improve infection control in laboratories lacking biosafety facilities. It is reported that bleach increase the sensitivity of smear microscopy through the digestion of mucus and debris in sputum and resulting in clearer microscopy field [19]. One of the notable disadvantages of bleach sedimentation is that a bleach treated sample cannot be used for mycobacterial culture, as the bleach kills *M. tuberculosis*.

Another recent development in smear microscopy is the advent of fluorescence microscopy (FM). Examination of sputum smears stained with Z-N requires on an average 5–10 minutes, consuming considerable working hours from the laboratories with limited resources. The newer alternative technique to Z-N smear microscopy, FM is known to increase the sensitivity (10% higher) when compared with Z-N microscopy methods while speeding up the whole process to consume much lesser time [20]. Fluorescent AFB can be seen at lower magnification than Z-N stained AFB. FM smears can be examined in a fraction (about 25%) of the time needed for Z-N smears as well. Recent development of simple FM systems based on light-emitting diodes (LED-FM) which have long life-spans, do not produce UV light, and have minimal power requirements could facilitate the implementation of FM in high burden and limited resources countries [21].

The use of sputum smear as a screening procedure for the diagnosis of pulmonary TB has recently been criticized following the finding by several large laboratories that up to 55% of specimens with positive smear failed to grow in culture while 30% are smear negative but culture positive [22,23]. The use of different smearing techniques varies from one laboratory to another. In most of the low and middle income countries the direct smear technique is used and only a few uses the concentrated method [23]. Though the resource needed to practice concentrated smearing technique remains a problem in many laboratories, it has been shown that concentration and liquefaction improve the sensitivity of the AFB smear microscopy and can contribute significantly to achieve better accuracy of diagnosis [24].

As microscopy is the mainstay of TB diagnosis in our country, in this study we wanted to compare the sensitivity obtained with smears for detection of AFB prepared directly from respiratory specimens (direct AFB smears) to that obtained with the parallel smears prepared from concentrate of the specimens (concentrated AFB smears) in our country context. This information may be of great help to the NTP in formulating effective TB control guidelines.

## Methods

### Specimen processing and culture

A total of 915 sputum specimens were aseptically collected from prisoners of the Dhaka Central Jail who were suspected to have pulmonary TB disease on the basis of their presenting symptoms. A suspect was defined as an individual if he/she had persistent cough for more than three weeks, and/or evening rise of temperature for more than two weeks, and/or body mass index (BMI) less than 16. A brief study questionnaire was used to identify the suspects among the inmates in Dhaka Central Jail, the largest prison in Bangladesh. The collected specimens were transported in specimen transportation box (cool box) to the Tuberculosis Laboratory at International Centre for Diarrhoeal Disease Research, Bangladesh (icddr,b) for AFB microscopy and culture.

Specimens were scored as saliva or sputum on the basis of visual examination. Suspects were requested to give another specimen in case of saliva specimens. This approach has been assessed as being feasible for the detection of specimens with a high probability of being positive a culture in a clinical setting. The use if this approach in a population-based screening programme outside the clinical setting is not to be recommended [25]. Direct smears were prepared by taking a small portion of the purulent part of the sputum with a sterile loop. The specimens were then processed by a standard NALC-NaOH digestion-decontamination method; briefly, an equal volume of 2% NaOH and 1.45% sodium citrate containing 0.5% NALC were added to each tube. The contents within the tubes were then mixed by vortexing and then incubated at room temperature for 15 minutes. The tubes were then shaken by hand at regular intervals. Phosphate-buffered saline (pH 6.8) were added up to 45 ml and then centrifuged at 3000X g for 15 minutes. The supernatant were carefully poured off, the resulting sediments were then re-suspended in 1.5 ml of phosphate-buffered saline, and the suspensions were used to prepare smear (concentrated smear) and to inoculate into Lowenstein-Jensen (L-J) culture media. Though the World Health Organization (WHO) recommended gold standard in TB diagnosis is culture of sputum in liquid media, in our study we have used culture in solid media (L-J) as gold standard as no liquid culture facility was available in our country during the study period.

### AFB smear and microscopy

Smears made from original specimens and/or from the concentrated specimens were air dried, heat fixed, and stained by the ZN staining technique. The stained slides were examined under oil immersion (1,000x lens objective), and they were reported negative when no AFB were seen in at least 100 microscopic fields. Smears were graded positive [17] for any of the following observations: when 1 to 9 AFB were seen in 100 microscopic fields (scored as scanty positive), when 10 to 99 AFB were seen in 100 fields (scored as 1+), when 1 to 10 AFB were seen per field in at least 50 fields (scored as 2+), and when more than 10 AFB were seen per field in at least 20 fields (scored as 3+). Direct smear and concentrated smears were examined separately by two laboratory technicians without knowing the results of each other. A total of 10% slides from both methods were re-examined by the laboratory supervisor and his classification was final in case of discrepancies.

All the specimens inoculated into L-J media were incubated at 37°C for 6 to 8 weeks in a vertical position for the better development of individual colonies. When small and buff colored colonies grew on LJ medium, the sample was considered as positive. Contaminated cultures (e.g. growth of moulds, and also those in which the medium had liquefied or turned dark green) were discarded.

The study protocol was approved by the Ethical Review Committee of International Centre for Dirrhoeal Disease Research, Bangladesh (icddr,b). All study subjects were enrolled in the study only after they had provided informed written consent.

### Data entry and analysis

All calculations were performed using SPSS, version 17.0 for Windows. Sensitivity and specificity were calculated for each diagnostic technique and expressed as percentages at 95% confidence intervals. The McNemar test was used to compare the sensitivity of the different tests.

## Results

Out of 915 specimens 74 (8.1%) were found to be AFB positive and 841 (91.9%) were found AFB negative by direct AFB smear. Among the positive specimens, 9 (1%) were scanty positive, 19 (2%) were 1+, 27 (3%) were 2+ and 19 (2%) were 3+. In contrast, 87 (9.5%) were found to be AFB positive and 828 (90.5%) were AFB negative when smear prepared from the concentrated specimens. Among the positive specimens 13 (2%), 18 (2%), 26 (3%), 30 (3%) were scanty positive, 1+, 2+ and 3+ respectively (Table 1).

**Table 1:** Comparison of quantization of AFB from direct and concentrated smear microscopy

**Direct smear results**	**No. of specimens with the following concentrated smear results:**					**Total**
	**Negative n (%)**	**Scanty n (%)**	**1 + n (%)**	**2 + n (%)**	**3 + n (%)**	
Negative	827 (98.3)	11 (1.3)	3 (0.4)			841
Scanty	1 (11.1)	2 (22.2)	6 (66.7)			9
1+			8 (42.1)	10 (52.6)	1 (5.3)	19
2+			1 (3.7)	16 (59.3)	10 (37.0)	27
3+					19 (100)	19
Total	828	13	18	26	30	915

Grading of AFB between the slides prepared from the same sample using direct and concentrated method was compared. Of the 915 specimens, 73 (8%) specimens were positive both on direct and concentrated methods, one sample was positive on direct microscopy but was negative on concentrated method. An extra 14 (1.5%) samples were positive on concentrated method which were negative on direct smear. More than 90% of the samples were found to be negative on both methods. Among 841 negative specimens on direct microscopy, 11 (1.3%) and 3 (0.4%) were found to be positive on concentrated methods. More than 66% of scanty, 52.6% of 1+ and 37.1% of 2+ AFB on direct microscopy were converted into 1+, 2+ and 3+ respectively on concentrated smear microscopy. No difference was found in case of high positive (3+) on both methods (Table 1).

Among the 915 specimens, 105 (11.5%) were found to be positive on culture. Considering 105 culture positive cases, 74 (70.5%) and 87 (82.9%) of them were AFB positive in direct and concentrated smear, respectively (Table 2). About 17.1% culture positive cases were found to be negative on both direct and concentrated AFB microscopy.

**Table 2:** Comparison of both microscopy methods with the gold standard culture method

**AFB microscopy**		**Gold standard +**	**Gold standard -**	**Total**	**Sensitivity % (95% CI)**	**Specificity % (95% CI)**
Direct	+	74	0	74	70.5 (60.8 – 79.0)	100 (99.5 – 100)
	-	31	810	841		
Concentration	+	87	0	87	82.9 (73.2 – 88.7)	100 (99.5 – 100)
	-	18	810	828		
Total		105	810	915		

The sensitivity of direct and concentrated smear microscopy was different when using positive culture result as the gold standard. Applying the McNemar *χ*^2^ test, the difference between sensitivities (71% versus 83%; p = 0.002) obtained by the two methods was found to be significant. These results showed that concentrated technique increases the sensitivity of microscopy up to 12% when performed with the same specimens. Among the 810 patients with negative cultures, the specificity (100%) was similar for both techniques.

## Discussion

This study showed that the use of the concentrated method for preparing smears for AFB microscopy increases sensitivity without a loss of specificity in identifying positive TB cases, compared to the direct method.

The specimen that was positive with the direct method but negative with the concentrated method was also found to be positive by culture. It was an uncommon phenomenon and theoretically it is difficult to explain such cases. It might have occurred due to inappropriate sample concentration and smear preparation, smear preparation from a negative sample accidentally, faulty staining process, or inappropriate microscopic observation.

Eight specimens were found to be positive in both direct and concentrated smear microscopy but negative in culture. From the clinical data, it was observed that these patients were taking anti-TB drugs while collecting the specimens. During treatment, only the dead/killed bacteria remain in the respiratory specimen (sputum) which were detected by microscopy but were unable to grow in L-J media.

A study conducted by Barez, et al. [26] showed that the sensitivity was almost similar in both methods as described 81.6% for direct method and 82.7% for the concentrated method. In another study, Cattamanchi et al. [27] failed to find a difference in sensitivity between direct and concentrated sputum smear microscopy, the calculated sensitivity of direct and concentrated smear microscopy was not significantly different (51% vs. 52%). In a similar study conducted by Peterson et al. [28] in two different laboratory settings (a tertiary-care laboratory and several local outpatients clinics) found that in a tertiary-care hospital the direct smear was significantly less sensitive than the concentrated smear (28% and 51%, respectively) and in the samples from outpatients of the Pacific islands the direct smear was less sensitive than that made from the concentrated specimen (82 versus 93%, respectively).

Despite some evidence that the concentrated method can be superior to the direct method, it is not being performed in peripheral TB laboratories in low-income countries, because of the following concerns: feasibility of centrifugation in settings with irregular power supply; limited human and financial resources; inadequate training capacity; lack of proper biosafety arrangements; and potential biohazard posed by centrifugation. In most resource poor countries like Bangladesh where TB is endemic, most of the microscopy centers are using direct smear microscopy with low sensitivity for the diagnosis of TB. However, in the light of our study findings, it may be recommend that concentrated smear microscopy can be used in place of conventional direct microscopy where appropriate facility is available, to achieve better diagnostic accuracy and ensure greater success of the TB control programmes. It may not be recommended as an ideal alternative to existing conventional microscopy considering the cost implications and other resource constraints involved with its implementation in the wide network of Bangladesh NTP, and other high TB burden countries. As alternative approaches like recently developed LED-FM microscopy and low cost sedimentation technique have shown to increase the sensitivity of microscopy without the involvement of much of resource, these can be considered to be incorporated in the national TB control activity framework rather than concentrated smear technique. However, for the district level laboratories where minimum biosafety arrangements can be made by putting in low cost biosafety cabinets and other constraints can be overcome, this concentrated smearing technique can be practiced to ensure better case detection of TB. There is still scope of further research to compare the outputs of concentrated smear technique and LED-FM in settings where appropriate resources are available. Research may also be conducted to find out the benefit of combining concentrated sample and fluorescent staining technique. As both these methods are already documented to increase the sensitivity of smear microscopy, their combination may have incremental effect and have the potential to increase the sensitivity of microscopy significantly.

## Conclusion

The study showed that concentrated AFB microscopy is more efficient to detect *M. tuberculosis* in respiratory specimens than direct AFB microscopy. Due to resource constraints it may not be suitable for implementing in the wide network of NTP microscopy centers thorough out the country but it may be considered for the district and other higher level TB diagnosis centers which have more resources available at their disposal to ensure greater success in TB case detection.

## Competing interests

The authors declare that no competing interests exist.

## Authors’ contributions

Conceived and designed the experiments: SB FvL. Performed the experiments: MKMU MRC. Analyzed the data: SB MTR RK. Wrote the paper: SB MKMU SA MTR FvL. Performed the field work: SB MTR. Facilitated data collection in the prison: MTR. All authors read and approved the final manuscript.
